# The effect of admitting fault versus shifting blame on expectations for others to do the same

**DOI:** 10.1371/journal.pone.0213276

**Published:** 2019-03-07

**Authors:** Elizabeth B. Lozano, Sean M. Laurent

**Affiliations:** Department of Psychology, University of Illinois Urbana-Champaign, Champaign, Illinois, United States of America; Rice University, UNITED STATES

## Abstract

A wealth of research has investigated how and why people cast blame. However, less is known about blame-*shifting* (i.e., blaming someone else for one’s own failures) and how exposure to a blame-shifting agent might lead to expectations that other agents will also shift blame. The present research tested whether exposure to a blame-shifting (versus responsibility-taking) agent would lead perceivers to expect a second, unrelated target to also shift blame. Contrary to our expectations, people expected greater blame-shifting after exposure to a *responsible* agent, particularly when perceivers were surprised by this reaction to failure. Discussion focuses on how people habitually expect some people to shift blame for their mishaps, and how expectancy violations when people act in unexpected ways predict the extent to which perceivers expect unrelated agents to also shift blame.

## Introduction

“*Men are only clever at shifting blame from their own shoulders to those of others*.”

-Titus Livius (59 BC–17 AD)

Titus Livius (“Livy”), a Roman historian, discussed blame shifting in the distant past, suggesting that even though people disapprove of it [1], this method of avoiding others’ censure is nothing new. Today, we continue to live in a culture where people remain motivated to avoid appearing blameworthy and quickly point their fingers at anyone but themselves when something goes wrong.

Although a substantial body of scholarly work has explored the nature of blame, as well as the cognitive and affective mechanisms that underlie people’s desire to blame and punish others for their actions (e.g., [2–9]), we know little about how exposure to blame-shifting agents impacts subsequent judgments, such as for other social targets. This is a worthwhile question to explore because blame shifting appears to be something social agents—particularly those in the public eye and concerned with their public image—engage in frequently when they publicly fail. For those who observe others’ attempts to shy away from blame, this may have unforeseen consequences, such as promoting a cynical expectation that other people will act in the same way, which can have consequences for how these others are socially evaluated. The present research explores this, investigating how exposure to leaders who shift blame for their own failures influences subsequent expectations that other agents will do the same.

### Failure and blame shifting

Because perceived weakness has the potential to ruin careers and reputations, it comes as no surprise that individuals seek to defend and bolster their self-images (e.g., [10, 11]) and monitor their public appearance in social settings [12]. In part, people are reluctant to admit they have failed because of a general desire to avoid negative social evaluation and disapproval from others (e.g., [13]). Thus, to save face when things go wrong, people will sometimes shift blame away from themselves by bringing attention to external causes [14], attempting to obscure their role in causing misfortune. This tendency may be especially true for individuals in the public eye (e.g., corporate leaders, politicians), as their failures are likely to be noticed by others and can cause repercussions such as loss of status, rank, or even employment [15, 16].

In the only paper to our knowledge examining downstream consequences from observing blame shifting, Fast and Tiedens [1] proposed a “blame contagion” hypothesis—the propensity for those who see others shifting blame to subsequently shift blame for their *own* failures. Specifically, the authors found that after exposure to a target who blamed someone else for a failure (versus accepting responsibility for it), perceivers were more likely to shift blame for their own personal failings. Several putative explanations for this effect were examined, such as mood and social learning, both of which were ruled out as causes of blame contagion. Another possibility that was examined regarded whether shifts in perceived acceptability of blaming might underlie the observed effect. However, regardless of experimental condition, participants rated blaming others for a failure as socially inappropriate. Although this explanation also failed to account for blame contagion, the finding highlights how blame shifting can negatively impact social evaluation, which is relevant to the present research. That is, if perceivers expect other, unrelated agents to shift blame for their failures after seeing another person shifting blame, this could taint their evaluation of these agents before they have even had a chance to respond. Moreover, even if a second agent does *not* shift blame, the expectation that they will could bias perceivers toward interpreting any explanation for a failure—even an acknowledgement of responsibility—as an attempt to deflect blame, resulting in negative evaluations of the agent.

Ultimately, Fast and Tiedens [1] showed that participants were more likely to develop a self-image protection goal after reading an article featuring an agent who blames others rather than taking responsibility, an idea that was supported by the elimination of the blame contagion effect when participants self-affirmed in an unrelated task before writing about a personal failure. This finding is intriguing but raises further questions. For example, if perceivers agree blame-shifting is socially undesirable, why then are they more likely to engage in this undesirable behavior themselves after seeing someone else do it? One possible answer provided the somewhat simple yet straightforward theoretical basis for the current work: although blame-shifting is seen as “wrong,” seeing someone do it also serves as a potent reminder that it can be employed as a successful strategy to avoid others’ disapproval, leading to unknowing use of the strategy in response to threat. Taking this one step further, observing blame-shifting (i.e., relative to taking responsibility) might make salient the idea that when someone is threatened, they will adopt this strategy because of its utility. This might then cause perceivers to interpret ambiguous information about a subsequently presented social target through a lens that is consistent with the adoption of a self-protection goal. In other words, seeing someone shifting blame might create an expectation that others will do the same.

### Behavioral expectancies and expectancy violations

Humans are evolutionarily hardwired to try and predict what others will or will not do (e.g., [17]). Perhaps because of this, people are surprisingly confident in the accuracy of their snap judgments, believing that their initial assumptions about how others will behave are correct [18]. However, judgments about others are often erroneous and based on heuristics that can backfire(e.g., [19]). This is particularly true when limited information is available for making judgments—a common state of affairs in social perception. Under uncertainty, people can only use those informational cues that are available [20] and often fall back on stereotypes as a way of easing the difficulty associated with decision-making [21].

In the context of judging how people will respond to failure, if stereotypes are available, they should serve as the basis for predicting behavioral responses. For example, if one thinks of CEOs as typically greedy, dishonest, and self-serving [22], then without further information, one might expect a CEO to shift blame for their own failures, rather than accepting responsibility. This should be particularly true when information about another person shifting blame has been recently presented and is therefore readily accessible in memory [23], and when the target being evaluated appears to belong to the same category as a previously evaluated target [24]. However, what might happen when an initially-presented target confounds rather than confirms stereotypes, violating expectations for behavior? Expectancy violation theory (EVT) [25], which describes how individuals respond to events that counter expectations and social norms in interpersonal contexts, might provide one clue. According to EVT, unexpected information receives more cognitive processing than expected information [26]. For example, behavior that is inconsistent with target-based expectancies [27] or stereotypes is often recalled better than consistent behavior.

In line with this framework, we originally hypothesized that expectancy-inconsistent behavior from an initially-presented target (i.e., who takes responsibility for a failure) would lead participants to question the accuracy of their stereotype (i.e., that CEOs will blame others for their failures), leading them to expect a subsequently presented target to also accept responsibility. On the other hand, when presented with a target whose behavior is consistent with an available stereotype, we expected that perceivers would assume a second target would do the same. Thus, compared with exposure to a person who defies expectations and takes responsibility, we expected that exposure to a blame-shifting target would cause perceivers to expect a new agent to do the same.

Ultimately, this is not what we found. The results of our first experiment, conducted with these expectations in mind, surprised us but also guided the development of a new hypothesis that we tested in two additional experiments: Blame-shifting may be so expected that any behavior serving to *disconfirm* it can lead to its greater expectation.

Although people anticipate that others will act in predictable ways, they also must be responsive to the presence of unexpected information that might demand the execution of a nonstereotyped behavioral response [28]. However, exposure to unexpected, stereotype-inconsistent behavior can also increase stereotype accessibility, leading to an expectation that others will behave more predictably. This might especially be the case when information about how a subsequently-presented target will respond is lacking or ambiguous, as it should force people to rely on available stereotypes (e.g., [29]). This might be particularly likely when perceivers have strong expectations about how an agent will behave.

Thus, when a target behaves stereotypically and normatively (i.e., for that group), people should not be particularly surprised, and they should continue to expect other similar targets to behave predictably, particularly when the behavior under consideration is in the same domain. On the other hand, when a target acts in a way that is surprisingly counter-stereotypical, people might expect a second target to behave more predictably. This suggests that the relationship between whether a first target confirms or violates a stereotype about blame shifting and judgments that a second target will shift blame could differ as a function of (i.e., interact with) the extent to which the first target’s behavior seems surprising.

Although we did not begin this work with this hypothesis in mind, it is consistent with other theoretical frameworks. For example, because people expect that others will act in ways that are consistent with available stereotypes, and these stereotypes are difficult to overcome, people who violate expectations—particularly in an extreme way—are treated as deviant exceptions to more general rules, allowing stereotypes to be maintained [30, 31]. In addition, because of the uncertainty that comes with expectancy violations [32], disconfirmation of stereotypes can evoke surprise [30, 33], perhaps resulting in an attempt to more accurately predict the behavior of a subsequently-presented target [32]. When little information is available to aid in this prediction, however, reliance on stereotypes may lead to the greatest accuracy [34], even if this means relying on the very stereotype that was recently violated.

### Experiment 1

Although we have already previewed how our own expectancies were violated, we describe our first experiment and hypotheses as originally conceived. Participants were presented with a fabricated news article about a CEO (John Kensington of “Global Health Services”) who failed in the launching of a website initiative for his company. The CEO was described as blaming independent contractors for the failed website launch (blame) or as taking full responsibility for the failure (responsibility). In a third condition, no mention of blaming others or taking responsibility was mentioned (control). After responding to questions about the extent to which this agent shifted blame and the extent to which participants’ expectancies for his behavior were confirmed or violated, participants read about a second, unrelated company failure and responded to questions about whether the CEO of the company would shift blame and whether people, in general, shift blame for their own failures. Our primary original hypothesis was that relative to control, participants who were exposed to an agent who shifted blame would expect greater blame shifting from the second agent, and that participants who were exposed to an agent who took responsibility would expect less blame shifting from a second agent. Our secondary (original) hypothesis was that participants in blame-shifting and responsibility-taking conditions would be respectively less and more surprised by the first agent’s behavior, and that surprise would mediate expectations that the second agent would shift blame.

### Method

#### Participants

A total of 179 participants (82 males, 96 females, 1 non-binary) from Amazon MTurk (AMT) participated in exchange for payment. Ages ranged from 19 to 81 (*M* = 35.12, *SD* = 11.62). On a 9-point scale asking, “In most matters (e.g., political, social, economic), where would you place yourself on the following scale (1 = *extremely liberal*, 9 *= extremely conservative*), the sample leaned slightly liberal (*M* = 4.16, *SD* = 2.41).

A sensitivity analysis showed that this sample size had sufficient power to detect an omnibus effect size $\eta_{p}^{2}$ = .050. Sample sizes for both experiments were determined *a priori* and data analysis did not begin until target sample sizes were reached. On the basis of unique AMT identifiers, we ensured that participants provided data for only one condition of one experiment. Informed consent was obtained from all participants prior to participation.

#### Procedure and measures

All stimuli are available on request and all fully de-identified data for all experiments are available at https://osf.io/29c7k. We report all manipulations and all variables used in both experiments. After agreeing to participate in a study described as a pretest of stimuli for future experiments, participants were randomly assigned to read one of three versions of a fabricated news article about a failed website launch (headed by “Global Health Services” CEO, John Kensington) that led to substantial shareholder losses. After describing the consequences of the website launch failure, participants in the blame condition read:

In a response to criticism, Mr. Kensington placed the blame squarely on independent contractors and their project leader, stating that the plan he proposed was not correctly implemented and changes made by the contractors were made without his approval… “Because of this, my team and I should not have to take the blame. The fault is theirs, not ours.”

Individuals in the responsibility condition read:

In a response to criticism, Mr. Kensington said that blame for the website failure rested solely with him, not with his team, and not with the independent contractors working on the website… “It is hard to admit, but I had a blind spot for my own plan. When you know you’ve made a mistake, it’s tempting to point the finger at someone else. But sometimes you just have to acknowledge that you’ve failed, hope investors will forgive you, and move on.”

Participants in the control condition read:

In a response to criticism, Mr. Kensington said that the website failure was not anyone’s fault. “Technology is hard to predict. You do the best you can, and our cost estimates were the best they could have been with the information we had. In the end, we think our product will be successful and profitable.”

After being presented with this information, participants indicated to what extent they thought the website launch was a failure (1 = *not at all a failure* to 7 = *very much a failure*). This measure served to ensure that across all conditions, participants perceived the event similarly as a failure [1]. To help disguise the nature of the research, participants were also asked how interesting and well-written the article was (1 = *not at all*, 7 = *extremely*; *r* = .59). Next, participants were asked the extent to which they thought the CEO was trying to avoid blame and protect his image (1 = *not at all*, 7 = *very much so*). These two items were averaged to create an index of perceived blame-shifting (*r* = .74) and served as a manipulation check. Three items assessed whether the CEO’s response to the failure violated or confirmed their expectancies (surprise; α = .87), all measured on 7-point scales (1 = *disagree completely*, 7 = *agree completely*): “The way the CEO responded to criticism is typical of businesspeople” (reverse-scored), “The way the CEO responded is surprising,” and “The way this CEO responded to criticism is unusual.” Two items also asked the extent to which people generally shift blame (*r* = .69): “In general, how often do people try to blame others for their own mistakes?” (1 = *almost never*, 7 = *almost always*) and “Rather than owning up to their mistakes, people tend to shift blame away from themselves” (1 = *disagree completely*, 7 = *agree completely*).

After providing responses, participants were presented with a second fictitious news article describing how a Quaker Oats plant had been recently flooded, causing millions of dollars of damage to the facility and products and also causing major supply disruptions that hurt ongoing revenues. Following this description, the vignette stated that Quaker’s CEO was expected to issue a statement in the coming days.

Participants were first asked how devastating the flood was for the Quaker Oats company (1 = *not at all*, 7 = *extremely*), followed by the same two questions used after the first article about how interesting and well-written the article was (*r* = .56). To capture the extent to which participants thought the CEO would shift blame (EBS-CEO), we asked six questions (*α* = .79): “How likely is it that Quaker’s CEO will try to protect his image?” “How likely is it that someone will be fired so that company executives have someone to blame?” (1 = *very unlikely*, 7 = *very likely*), “Quaker’s CEO will probably try to shift the blame for the failure away from himself,” “Company executives will find someone—anyone but them—they can blame for what happened,” “Probably, Quaker’s CEO will respond in a way that is typical of businesspeople,” and, “I doubt that anything the CEO says would surprise me” (1 = *disagree completely*, 7 = *agree completely*). Two items measured general expectations for blame-shifting (EBS-general; *r* = .74): “In general, how often do people try to blame others for their own mistakes? (1 = *almost never*, 7 = *almost always*) and, “Rather than owning up to their own mistakes, people tend to shift the blame away from themselves” (1 = *disagree completely*, 7 = *agree completely*). After responding to these questions, participants provided demographic information and were thanked for their participation.

### Results and discussion

#### Story 1

All reported ANOVAs were based on 2, 176 *df*. No differences in perception of the website launch as a failure or quality of the story emerged as a function of condition, respectively, *F*s = 0.20 and 2.03, *p*s = .819 and .134. As expected, perceived blame-shifting was significantly impacted by condition (*F* = 115.02, *p* < .001, $\eta_{p}^{2}$ = .57), with reported blame-shifting highest in the blame condition (*M* = 6.28, *SD* = 0.98), lowest in the responsibility condition (*M* = 2.62, *SD* = 1.66), and intermediate (but still somewhat high) in the control condition (*M* = 5.27, *SD* = 1.32). Post-hoc Tukey tests showed that all means significantly differed from one another, *p*s < .001. Also as expected, surprise was affected by condition, *F* = 56.81, *p* < .001, $\eta_{p}^{2}$ = .39. Mean surprise in the responsibility condition (*M* = 5.25, *SD* = 1.45) was higher than in the blame (*M* = 2.99, *SD* = 1.38) and control (*M* = 2.67, *SD* = 1.44) conditions, *p*s < .001. However, blame and control conditions did not differ on surprise, *p* = .426. This result on surprise suggests that participants might have perceived the control CEO’s refusal to acknowledge responsibility for the failure (i.e., saying no one was to blame) as a passive attempt to deflect blame and protect his image, similar to the more active strategy used in the blame-shifting condition. General expectations for blame-shifting were not significantly impacted by condition, *F* = 2.03, *p* = .135.

#### Story 2

No significant condition-based differences emerged for how devastating the flood at Quaker was (*F* = 1.55, *p* = .216) or how interesting/good the story was, *F* = 1.21, *p* = .300. Although, as expected, there was a significant effect of condition on EBS-CEO *F* = 5.17, *p* = .007, $\eta_{p}^{2}$ = .06, the ordering of means ran counter to our (original) primary hypothesis. Post hoc Tukey tests showed that rather than being lower than blame or control conditions, EBS-CEO (i.e., expected blame shifting from the agent in the second story) was *highest* in the responsibility condition (*M =* 5.14, *SD* = 1.01), and significantly higher than in the blame (*M* = 4.56, *SD* = 1.06) and control (*M* = 4.63, *SD* = 1.12) conditions, respectively, *p*s = .010 and.025. Also counter to our initial prediction, blame and control did not differ, *p* = .934. This further suggests that participants in the control condition saw the CEO’s refusal to accept blame as another strategy for shifting blame away from himself without directly placing it on someone else. Similarly, there was a marginally significant effect of condition on EBS-general, *F* = 2.74, *p* = .068, $\eta_{p}^{2}$ = .030. The only marginally significant post hoc comparison was between the responsibility (*M* = 5.66, *SD* = 1.03) and control conditions (control *M* = 5.21, *SD* = 1.04; blame *M* = 5.43, SD = 1.06), *p* = .053. No other pairwise comparisons were significant, *p*s > .464.

Initially, the present work was based on Fast and Tiedens [1] somewhat paradoxical finding that after exposure to a blame-shifting target, participants are more likely to also shift blame for their own failures—even though they agree that this is not an acceptable social behavior. That is, if despite indicating that shifting blame is wrong, people are more likely to shift blame themselves after exposure to a stereotype-confirming (i.e., blame-shifting) target, we thought that through a process of expectancy confirmation, this blame-contagion effect would extend beyond the self and impact how people evaluate *other* social targets. Similarly, we expected that when an initial target violates expectancies by taking responsibility for their failure, people might revise their original stereotypes and expect a second target to also take responsibility. Moreover, we predicted that condition-based differences in expected blame shifting would be mediated by the extent to which the initially-presented targets’ behavior was surprising to participants.

Instead, we found that after exposure to an agent who acted counter-stereotypically (i.e., by taking responsibility), people expected a second agent to blame others for a failure even *more* than when they were exposed to an agent who blamed others, and that there was no mean difference in EBS for the second agent when comparing blame and control conditions. Although the latter finding is less surprising because the CEO in the control condition might have also been seen as trying to protect his self-image (i.e., as having tried to passively shift blame), the primary finding puzzled us. Yet, in trying to understand this result, we arrived at a new and potentially more interesting hypothesis: People *expect* powerful agents to shift the blame so much that when they do not, it only heightens the expectancy that other powerful agents *will*. That is, to the extent that an agent’s behavior seems counter-stereotypical, perceivers might subtype them as a deviant (e.g., [30]), allowing them to retain their original stereotype and increasing the likelihood that the stereotype will applied in trying to predict the behavior of a new target that is similar to the first. If true, then when participants are particularly surprised by a counter-stereotypical target’s behavior, they should seek greater accuracy in predicting the behavior of another target [32] and be especially likely to apply the violated stereotype to this target when little other information is available. This suggests an interaction between surprise and target type on subsequent expectations for blame shifting should be found. To test this, we examined the interaction of condition with surprise by the first agent’s actions to predict EBS for the second agent.

#### Moderation

**Fig 1:**
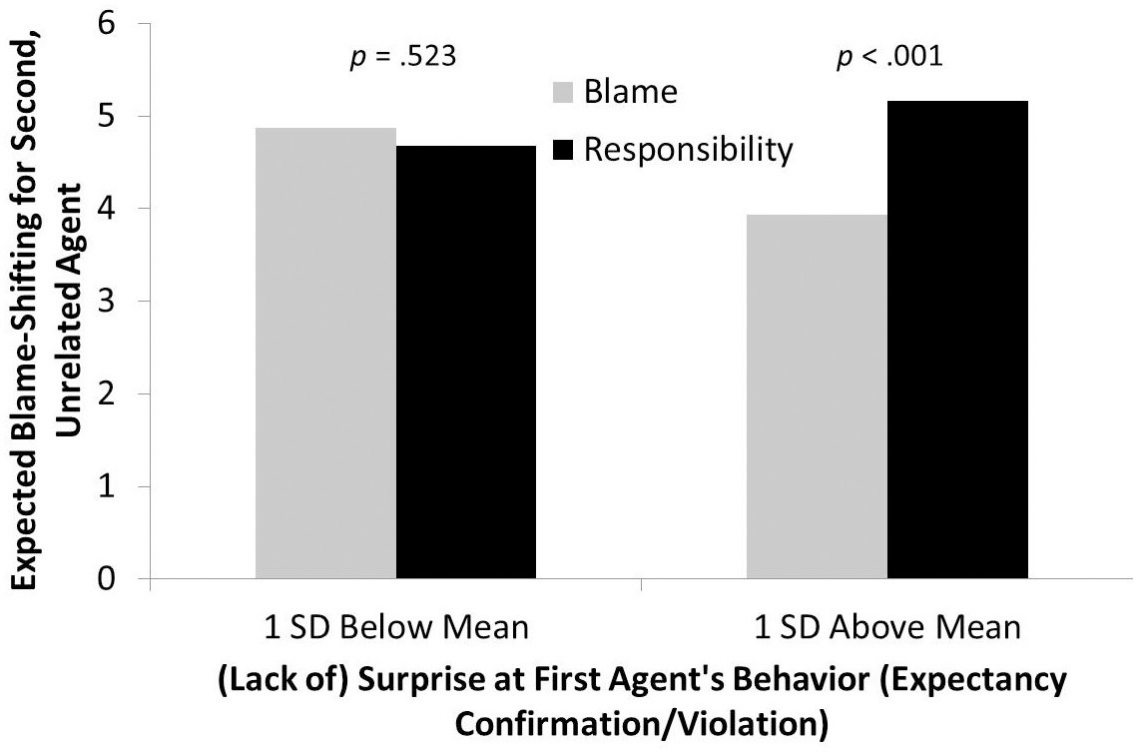
Experiment 1 EBS. Expected blame-shifting for Quaker CEO (second agent) as a function of experimental condition and surprise at first agent’s behavior.

Hierarchical regression was used, with condition (dummy-coded) and surprise (mean-centered) entered in a first block and their interaction entered in a second block [35]. Because our main interest regarded whether differences in EBS-CEO that emerged as a function of being in the responsibility versus the blame condition changed as a function of surprise (i.e., we had no hypothesis for control), the control condition was excluded from this analysis. Condition was coded as responsibility = 0 and blame = 1. Consistent with our revised hypothesis, the interaction between condition and EBS-CEO was significant, *b* = -0.39, Δ*R*^2^ = .07, *ΔF*(1, 113) = 8.74, *p* = .004. As can be seen in Fig 1, when people were particularly surprised by the first CEO’s behavior, they expected the second agent to shift blame more in the responsibility condition relative to the blame condition. However, below the mean on surprise, no significant difference in EBS-CEO was found. It is worth noting that when an additional dummy-code was added to include control in the analysis, the omnibus interaction was significant (Δ*R*^2^ = .042, Δ*F*(2, 173) = 4.07, *p* = .019) and the interaction coefficient for surprise x responsibility versus blame was unchanged, *b* = -.39, *p* = .005. The interaction of surprise x the contrast of the responsibility condition versus control was marginally significant (*b* = -.22, *p* = .097).

In a second, exploratory analysis we examined the interaction of condition and surprise on general expectations for people to shift blame. Here, the effect was even stronger, *b* = -.60, Δ*R*^2^ = .16, *F*(1, 113) = 22.27, *p* < .001. At one *SD* above the mean on surprise, people in the responsibility condition (*M* = 5.71) expected others to shift blame to a greater extent than in the blame condition (*M* = 4.69), *p* = .001. One *SD* below the mean on surprise, this reversed (responsibility *M* = 4.64, blame *M* = 5.81), *p* = .002. This suggests that to the extent that people view a powerful agent’s responsibility-taking behavior as counter-stereotypical and surprising, they are more apt to expect the opposite from everyone, but when they do not view this behavior as anything special, these expectations for others are diminished. On the other hand, when a blame-shifting agent’s behavior does not seem surprising (e.g., because it confirms expectations), people expect similar bad behavior from others; when it *is* particularly surprising, people believe that others are not as likely to do the same.

### Experiment 2

Experiment 2 had two goals: First, we wanted to replicate the primary findings of Experiment 1 to provide another test of our revised hypothesis. Second, to increase generalizability, we wanted to see if the same effect would emerge using a different second agent who is powerful but is not another CEO, and should therefore not have other attributes in common with a CEO. Participants in blame and responsibility conditions were exposed to the same initial fabricated article as in Experiment 1 (i.e., a failed website launch), but were then exposed to a new second news story about a medical doctor whose actions led to the death of a patient during a relatively routine cosmetic surgery procedure. In this story, details were left ambiguous regarding whether the death of the patient was a result of complications from anesthesia or negligence. In addition to variables intended to measure perceived blame-shifting, surprise, and expected blame-shifting, we also included exploratory items tapping evaluation of the first agent (e.g., as a good business leader) and a measure of blame for the second agent.

Our first hypothesis was that relative to the blame condition, participants in the responsibility condition would be more surprised by the first agent’s behavior. Our second hypothesis was that participants in the responsibility condition would expect more blame-shifting (i.e., greater EBS) from the second agent than participants in the blame condition. Finally, we hypothesized that surprise and condition would interact such that when surprise at the first agent’s behavior was higher, EBS would be higher in the responsibility than in the blame condition, but that when surprise was lower, no significant differences in EBS would be found (i.e., replicating the effect from Experiment 1).

Representing two secondary predictions that were more exploratory, we expected (a) that people would evaluate the (first) blame-shifting agent more negatively, and (b) that the second agent would be perceived as equally culpable across conditions. That is, people should prefer the responsibility-taking agent because they believe that blame shifting is wrong, but despite heightened expectations in this condition for a second agent to shift blame, ambiguity in the second story about why the patient died should lead to similar judgments of the physician’s actual blameworthiness across conditions.

### Method

#### Participants

A total of 182 participants (81 males, 99 females, 2 non-disclosed) from Amazon MTurk participated in exchange for a small wage. Ages ranged from 19 to 70 (*M* = 36.48, *SD* = 11.89). On the same 9-point ideology scale used in Experiment 1, the sample again leaned slightly liberal (*M* = 4.27, *SD* = 2.18).

#### Procedure and measures

Participants were again told that the purpose of the study was to pre-test materials for future research and were randomly assigned to a blame-shifting (blame) or responsibility-taking (responsibility) condition, where they were exposed to the same fabricated first article used in Experiment 1. No control condition was used. After reading the first story about the website initiative at Global Health, participants responded to the same two questions used in Experiment 1 about avoidance of blame and image protection (perceived blame-shifting, *r* = .83) and were asked two questions assessing their surprise by the CEO’s behavior (*r* = .55): “The way the CEO responded was…” (1 = *not at all surprising*, 7 = *very surprising*) and, “The way the CEO responded was…” (1 = *not what I would expect from a CEO*, 7 = *exactly what I would expect from a CEO*; reverse-scored).

Eleven items also asked for participants evaluations of the CEO. An exploratory principal components analysis showed that seven of these items loaded on one component and four loaded on a second component. Further exploratory analyses showed that associations among the four items loading on the second component (i.e., whether the CEO would or should fight to preserve his reputation, whether other CEOs would respond similarly, and whether CEOs should try to do what is best for themselves or best for shareholders) differed within conditions, which would make any analyses using them difficult to interpret. These four items were therefore excluded from further analyses. A second principal components analysis using only the seven retained items revealed that they continued to load together on a single component explaining 69.32% of their variance, and were therefore averaged to create a measure of evaluation of the CEO (higher numbers indicate a more positive evaluation; α = .93): “In response to criticism, this CEO did the right thing,” “The CEO was willing to harm his reputation for the good of the company,” “This CEO provides a good example of how to get ahead in business” (1 = *disagree completely*, 7 = *agree completely*), “This CEO is a … leader” (1 = *weak*, 7 = *strong*), “In business, this CEO is a…” (1 = *loser*, 7 = *winner*), “By responding as he did, the CEO was trying to help…” (1 = *himself*, 7 = *the shareholders*), and “How much ability does this CEO have to execute a plan?” (1 = *very low ability*, 7 = *very high ability*).

Next, participants were presented with a second fabricated article about a patient (“Ms. Ackerman”) who died during a routine facelift procedure. After a brief introduction, it read as follows:

Minutes after a doctor administered local anesthesia, Ackerman’s body jerked violently and the oxygen in her blood plunged. By the time the clinic called an ambulance, the woman had died. Because this was a standard procedure, Ackerman was not hooked up to any continuous-monitoring equipment and no anesthesiologist was present, so the medical staff did not know immediately how little oxygen she was getting.

In response to the incident, an anonymous source commented, “The patient experienced an unknown complication in response to the anesthesia that was administered. We couldn’t have expected this to happen. Sometimes adverse drug reactions are hard to predict. You do the best you can, and our decisions were the best they could have been with the information we had.

The physician who conducted the procedure, Dr. James Connell, is expected to issue a statement in the coming day.

Participants then responded to two questions about expected blame-shifting by the doctor (EBS-doctor; *r* = .70): “Dr. Connell (the physician in charge of the facelift procedure) will…” “try to protect his image” and, “try to shift the blame for the failure away from himself” (1 = *disagree completely*, 7 = *agree completely*). Using the same scale, a single question asked about expected blame-shifting in general (EBS-general): “Rather than owning up to their own mistakes, people tend to shift blame away from themselves.” One question asked: “In response to what happened, nothing Dr. Connell might say would surprise me” (1 = *totally disagree*, 7 = *totally agree*). Finally, two questions assessed the extent to which the doctor should be blamed for Ms. Ackerman’s death (*r* = .73): “Who deserves the most blame for the failed procedure?” (1 = *Ms*. *Ackerman (the patient)*, 7 = *Dr*. *Connell (the physician)*) and, “Lifestyle Lift physician, Dr. Connell, deserves to be sued for negligence” (1 = *totally disagree*, 7 *= totally agree*).

### Results

#### Story 1

All *t-*tests were based on 180 *df* and all *t*s and associated effect sizes are reported as absolute values. As expected, participants in the blame condition thought the CEO shifted blame (*M* = 6.20, *SD* = 0.92) to a greater extent than participants in the responsibility condition (*M* = 2.46, *SD* = 1.53), *t* = 20.11, *p* < .001, *d* = 2.96. Supporting our first hypothesis, participants were more surprised by the behavior of the responsible CEO (*M* = 4.85, *SD* = 1.43) than by the behavior of the blame-shifting CEO (*M* = 3.21, *SD* = 1.60), *t* = 7.27, *p* < .001, *d* = 1.08. In addition, the responsibility-taking CEO was evaluated more positively (*M* = 5.37, *SD* = 0.95) than the blame-shifting CEO (*M* = 2.92, *SD* = 1.16), *t* = 15.49, *p* < .001, *d* = 2.31.

#### Story 2

Replicating Experiment 1 and supporting our second hypothesis, EBS-doctor was higher in the responsibility condition (*M* = 5.88, *SD* = 1.03) than in the blame condition (*M* = 5.49, *SD* = 1.27), *t* = 2.25, *p* = .026, *d* = 0.34. Yet, despite indicating that the physician would blame others more for the patient’s death, participants in the responsibility condition (*M* = 5.26, *SD* = 1.28) did not blame the physician significantly more than they did in the blame condition (*M* = 4.96, *SD* = 1.33; *t* = 1.54, *p* = .125). Participants also indicated that they would not be any more surprised by what he might say (responsibility *M* = 4.61, *SD* = 1.73; blame *M* = 4.49, *SD* = 1.70; *t* = 0.49, *p* = .625) and did not think that in general (EBS-general), people shift blame to a greater extent (responsibility *M* = 5.83, *SD* = 1.09; blame *M* = 5.66, *SD* = 1.12), *t =* 1.04, *p* = .301.

#### Moderation

**Fig 2:**
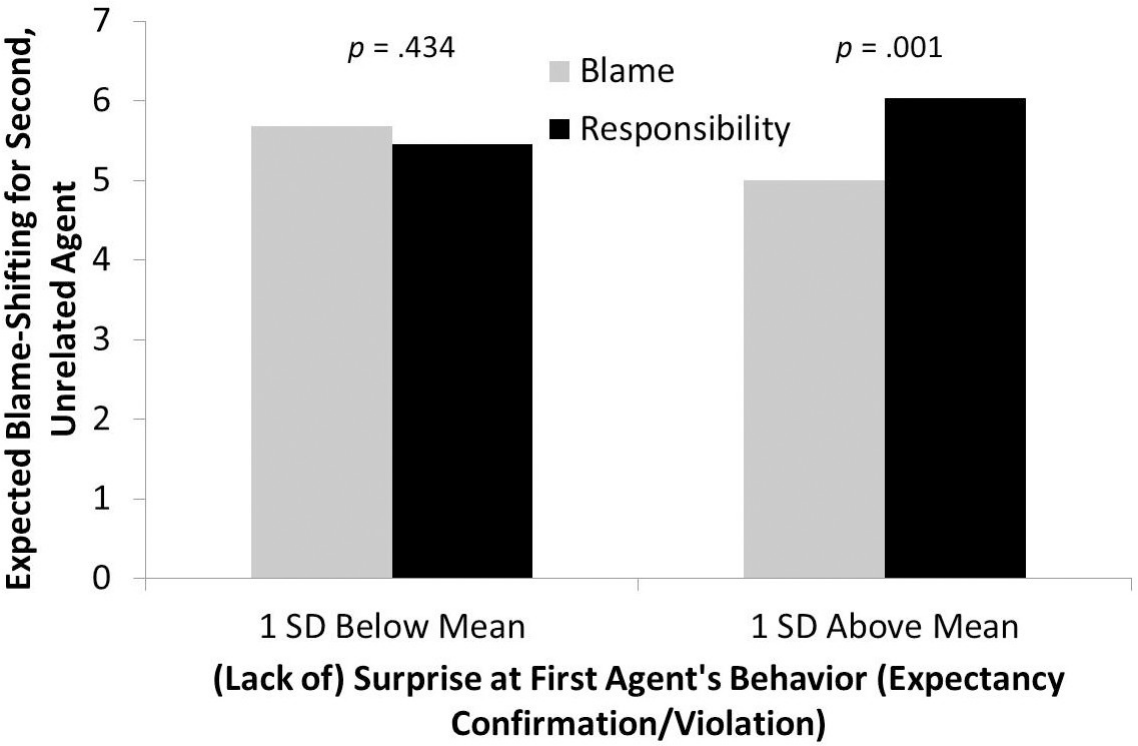
Experiment 2 EBS. Expected blame-shifting for surgeon (second agent) as a function of experimental condition and surprise at first agent’s behavior.

Also replicating Experiment 1 and confirming our third hypothesis, the interaction between condition and surprise was significant, *b* = -0.36, Δ*R*^2^ = .054, *ΔF*(1, 178) = 10.46, *p* = .001. As can be seen in Fig 2, one *SD* below the mean on surprise, there was no significant difference in expected blame shifting between blame and responsibility conditions; one *SD* above the mean, the responsibility condition was significantly higher on EBS-doctor than the blame condition.

Although the main effect of condition on EBS-general was not significant, we examined again whether surprise and condition would interact to predict it. The interaction was significant, *b* = -.40, Δ*R*^2^ = .07, *ΔF*(1, 178) = 14.23, *p* < .001. Replicating Experiment 1, one *SD* below the mean on surprise, the blame condition was significantly higher on EBS-general than the responsibility condition, *p* = .049. One *SD* above the mean, this reversed, *p* = .001. In an exploratory analysis, we examined whether blame or surprise by what the surgeon might say were impacted by the same interaction. Neither variable was: respectively, *p*s = .619 and.477.

### Discussion

Replicating the finding from Experiment 1 and consistent with our revised hypothesis, presenting participants with story about a CEO who took responsibility for a website launch (versus blaming others for the failure) led participants to expect greater blame-shifting from a second, unrelated agent. In this case, the second agent was not another CEO but was a surgeon whose patient died during routine cosmetic surgery, showing that the effect can generalize to a different target in a different profession. Also replicating Experiment 1, greater surprise by the behavior of the responsibility-taking agent in a first story translated into even greater expectations for the second agent to shift the blame, but at lower levels of surprise, no condition-based effect on blame shifting was found. Moreover, although there was no total effect of condition on a general measure of expected blame-shifting, the same interaction found in Experiment 1 emerged again here. Below the mean on surprise, participants in the blame condition expected others to shift blame to a greater extent than in the responsibility condition. Above the mean on surprise, this reversed.

### Experiments 3a and 3b

The unexpected findings of Experiment 1 led to the generation of a revised hypothesis, and a similar pattern of results in Experiment 2 confirmed the finding. However, our revised hypothesis was built on a theoretical foundation that assumed people *expect* business leaders to shift the blame. Although this assumption seemed warranted, we also realized it would be helpful to provide evidence that people hold stereotypes about business leaders as blame shifters. Thus, we conducted a simple preregistered experiment to find out whether people agree that business leaders habitually shift blame. In addition, we conducted a separate study that asked whether people hold stereotyped beliefs that politicians habitually shift blame. This was in anticipation of conducting one final study to address a few issues that arose in Experiments 1 and 2 and to test whether our findings would generalize to a new domain. Our sole hypothesis in both studies was that people would believe that business leaders and politicians typically shift blame for their failures, rather than taking responsibility for them. Both of these experiments were preregistered (available at http://aspredicted.org/blind.php?x=jz6c98) and the preregistration includes all hypotheses and our data analysis plan.

### Method

#### Participants

Across both experiments (Experiment 3a (CEO), *n* = 60; Experiment 3b (Politician) *n* = 61), a total of 121 participants (59 males, 62 females) from Amazon MTurk participated in exchange for a small wage. Ages ranged from 18 to 71 (*M* = 36.01, *SD* = 12.74). On the same 9-point ideology scale used in Experiments 1 and 2, the sample again leaned slightly liberal (*M* = 4.40, *SD* = 2.32).

#### Procedure and measures

After consenting to participate, participants were instructed to think about how business leaders (CEO’s; Experiment 3a) or politicians (Experiment 3b) typically respond to failure and were provided with an example of what a failure might be (i.e., starting a program and losing profits or failing to adequately safeguard against a disaster; trying to achieve a political goal and failing). Participants were then asked how they think business leaders (CEO’s) or politicians would typically respond to failures. In each experiment, participants responded to four questions (Experiment 3a α = .85; Experiment 3b α = .82): “(CEO’s) (Politicians)” “almost always shift blame away from themselves for failures,” “usually blame anyone but themselves for a failure,” “almost always take the blame for failures” (reverse-scored), and “usually accept responsibility for failures” (reverse-scored) (1 = *totally disagree*, 4 = *neither agree nor disagree*, 7 = *totally agree*).

### Results and discussion

To test whether participants held stereotypes about business leaders and/or politicians, we conducted single-sample *t*-tests against scale midpoints in each experiment. In both experiments, average responses were significantly above the scale midpoints: Experiment 3a (CEO) *M* = 4.56, *SD* = 1.46, *t*(59) = 2.99, *p* = .004, *d* = 0.38; Experiment 3b (Politician) *M* = 5.63, *SD* = 1.03, *t*(60) = 12.29, *p* < .001, *d* = 1.58. Given that average responses were significantly above both scale midpoints (i.e., indicating agreement with the statements rather than disagreement or ambivalence), this suggests that people habitually expect both business leaders (CEO’s) and politicians to shift blame in response to the experience of failure.

### Experiment 4

One final study was conducted to examine whether the effects we found in Experiments 1 and 2 (i.e., on expected blame shifting for a second, unrelated target after exposure to a blame-shifting or responsibility-taking target) would emerge when participants were exposed to a new blame-shifting target that was not a business leader. In Experiment 4, we decided to use politicians for both targets.

Experiment 4 also addressed two other concerns. First, in Experiments 1 and 2, the blame-shifting agents were both men. Second, in the responsibility conditions, several pieces of information were included that did not appear the blame condition, making it harder to draw firm conclusions. For example, the responsibility-taking agent acknowledged his failure (“when you know you’ve made a mistake”), expressed a descriptive norm regarding expectations for blame shifting (“it’s tempting to point the finger”), and asked for forgiveness (“…hope investigators will forgive you”). None of these sentiments were expressed in the blame-shifting condition. Experiment 4 addressed these potential concerns. Specifically, participants read about a female (rather than male) agent who shifted blame. Also, agents in both conditions acknowledged the failure, neither asked for forgiveness (although both asked their audiences for their votes), and no descriptive norms about expectations for blame shifting were mentioned in the responsibility condition. Hypotheses were the same as in Experiment 2. This experiment was also preregistered (available at http://aspredicted.org/blind.php?x=as6g2f) and includes all hypotheses and plans for data analysis.

### Method

#### Participants

A total of 200 participants (86 males, 114 females) from Amazon MTurk participated in exchange for a small wage. Ages ranged from 20 to 72 (*M* = 40.97, *SD* = 13.24). On the same 9-point ideology scale used in Experiments 1 and 2, the sample again leaned slightly liberal (*M* = 3.91, *SD* = 2.23).

#### Procedure and measures

After consenting to participate, participants were told that we were interested in how people respond to politicians when they talk about their failures to achieve political goals. They were instructed that they would read two news articles about politicians who had experienced failures and answer a few questions about each one. Next, participants were presented with an ostensibly real news story about a female politician (no political party affiliations were mentioned and her name was masked; this is denoted below with three X’s) who failed to enact a jobs plan she campaigned and won on. She either blamed this on her political rivals or took responsibility. Information appearing only in the blame condition is bolded; information only appearing in the responsibility condition is bolded and inside parentheses:

At a recent rally for re-election, XXX representative XXX XXX **blamed her political rivals for blocking a** (**took responsibility for failing to pass the**) jobs plan she campaigned (and won) on. As we have reported previously, Ms. XXX has been facing harsh criticism in the local press. During the rally, representative XXX told her supporters, “I ran for this office with a promise to bring jobs to our local communities, to give every person who lives here the chance to work at a good job, to earn a good living, in order to provide for yourselves and your children. However, as we all know, things haven’t gotten better for many of you. You’re still hurting. There aren’t enough jobs, and the jobs people have don’t pay what they should.

I ran on this promise, and every person in this room knows I have done everything I can to fulfill my promise to you. But, as I’m sure you all know, **my opponents have tried to block my plan at every opportunity** (**getting my plan passed has been harder than I had expected**). If you are looking for someone to blame, you don’t have to look far—**the blame belongs to my opponents** (**the blame is mine alone**). Fixing things won’t be easy, I know that. It will take even more dedication and hard work on my part, working for all the people of this state. But if you re-elect me, I vow to finish what I started the first time you elected me. I vow to get good-paying jobs for every person in this room, for every person in this state, who wants one.

Next, participants were asked three filler questions about the story. One question asked about her failure: “To what extent was the candidate’s attempt to bring new jobs to her community a failure?” (1 = *not at all a failure*, 7 = *very much a failure*). Two questions then asked about how interesting and well written the story was (*r* = .64) (1 = *not at all*, 7 = *extremely*). Two questions checked the manipulation, asking about the extent to which the candidate was trying to protect her image and avoid being blamed (perceived blame shifting; *r* = .66) (1 = *not at all*, 7 = *very much so*). Two questions asked about expectancy confirmation/violation (surprise; *r* = .37): “The way the candidate responded was…” (1 = *not at all surprising*, 7 = *very surprising*) and (1 = *not at all what I would expect from a politician*, 7 = *exactly what I would expect from a politician*; reverse-scored). We note that because of the lower than expected correlation between the two surprise items, we also conducted additional unplanned analyses using each variable. Results from these analyses were fully consistent with the analyses we report here. Finally, one question (likable) asked: “How likable is the politician you read about?” (1 = *not at all likable*, 7 = *extremely likable*).

Next, participants read a second story about a female governor whose office was dealing with a corruption scandal after the governor had been elected to office by a thin margin on an “anti-corruption” platform. Examples of the alleged corruption were provided (e.g., members of her office receiving gifts such as paid vacations and cash bribes) and the governor’s efforts to clean up corruption were described by critics as a complete failure. The governor herself was described as not suspected of any wrongdoing.

Participants then responded to a single question asking about the extent to which the governor’s goal of stopping corruption was a failure (1 = *not at all*, 7 = *very much*) and how interesting and well-written the article was (*r* = .65). Next, participants answered five questions about their expectations that the governor would shift blame for the failure (EBS-Governor; α = .87): “Do you think the governor will try and protect her image by shifting blame for corruption in her office?” (1 = *absolutely not*, 7 = *absolutely*); “To what extent will the governor try to avoid blame for the corruption scandal?” (1 = *not at all*, 7 = *completely*); “How likely is it that the governor will try and avoid blame?” and “How likely is it that the governor will blame someone else for her subordinates’ actions?” (1 = *not at all likely*, 7 = *very likely*); “How likely is it that the governor will take responsibility for the corruption in her office?” (1 = *not at all likely*, 7 *= very likely*; reverse-scored). One question asked about general blame shifting (EBS-General): “Rather than owning up to their own mistakes, people tend to shift the blame away from themselves” (1 = *totally disagree*, 7 = *totally agree*). Finally, one question asked (EBS-Politicians): “Typically, politicians _____ for their failures” (1 = *shift blame*, 7 = *take responsibility*; reverse-scored).

### Results and discussion

#### Story 1

All *t-*tests were based on 198 *df* and all *t*s and associated effect sizes are reported as absolute values. No difference emerged in the extent to which the first story was seen as interesting and well-written, *t* = 0.87, *p* = .386. However, there was a small and unexpected marginally significant difference in the extent to which participants believed the politician’s inability to enact her jobs platform was a failure. Participants in the responsibility condition (*M* = 6.11, *SD* = 0.99) thought it was a slightly greater failure than participants in the blame-shifting condition (*M* = 5.81, *SD* = 1.17), *t* = 1.95, *p* = .053, *d* = 0.27. Although this effect was quite small and only marginally significant, it does hint at the possibility that shifting blame can diminish the perception of failure (i.e., work as an impression management strategy). However, given that no such difference emerged in Experiments 1 and 2, this finding should be treated very cautiously. Confirming that the manipulation worked, participants in the blame-shifting condition (*M* = 6.35, *SD* = 0.87) believed the politician shifted blame to a larger extent than participants in the responsibility condition (*M* = 3.63, *SD* = 1.57), *t* = 15.18, *p* < .001, *d* = 2.14. Also as expected, participants were less surprised by the behavior of the blame-shifting politician (*M* = 2.35, *SD* = 1.20) as compared with the responsibility-taking politician (*M* = 3.93, *SD* = 1.52), *t* = 8.19, *p* < .001, *d* = 1.15. Finally, consistent with the idea that people do not like blame shifting, participants liked the responsibility-taking politician (*M* = 4.96, *SD* = 1.25) more than the blame-shifting politician (*M* = 4.03, *SD* = 1.63), *t* = 4.53, *p* < .001, *d* = 0.64.

#### Story 2

No significant differences emerged in the extent to which the governor had failed to stop corruption or in the extent to which the story was perceived as interesting or well written, *t*s < 1.61, *p*s > .10. Also, contrary to our hypotheses, no main effect emerged for EBS-Governor (*t* = 1.51, *p* = .132), EBS-General (*t* = 0.75, *p* = .455), or EBS-Politicians (*t* = 0.96, *p* = .338). If anything, there was a slight trend toward expecting *more* blame from the second, unrelated politician in the blame-shifting condition (*M* = 5.72, *SD* = 1.08) than in the responsibility-taking condition (*M* = 5.49, *SD* = 1.05). This was somewhat unexpected, but speculatively, the lowered expectation for blame shifting might have resulted from the somewhat diminished surprise participants reported at the behavior of the politician in the responsibility-taking condition. That is, relative to in Experiments 1 and 2, where surprise in the responsibility conditions was somewhat high, in the current experiment, surprise hovered near the scale midpoint. Again speculatively, participants may have been less surprised in the responsibility-taking condition because even though the politician (surprisingly) took responsibility for her failure to secure jobs for her constituents, her behavior was in other ways not surprising at all. That is, most of what she said was consistent with the behavior of a typical politician who is trying to get re-elected. Thus, relative to the behavior of the agents in Experiments 1 and 2, participants may have de-emphasized the unusual aspect of her behavior (i.e., taking responsibility) and focused on those aspects that were less unusual (i.e., trying to excite her base), particularly since the questions about surprise were quite general, asking only about “her response.”

#### Moderation

**Fig 3:**
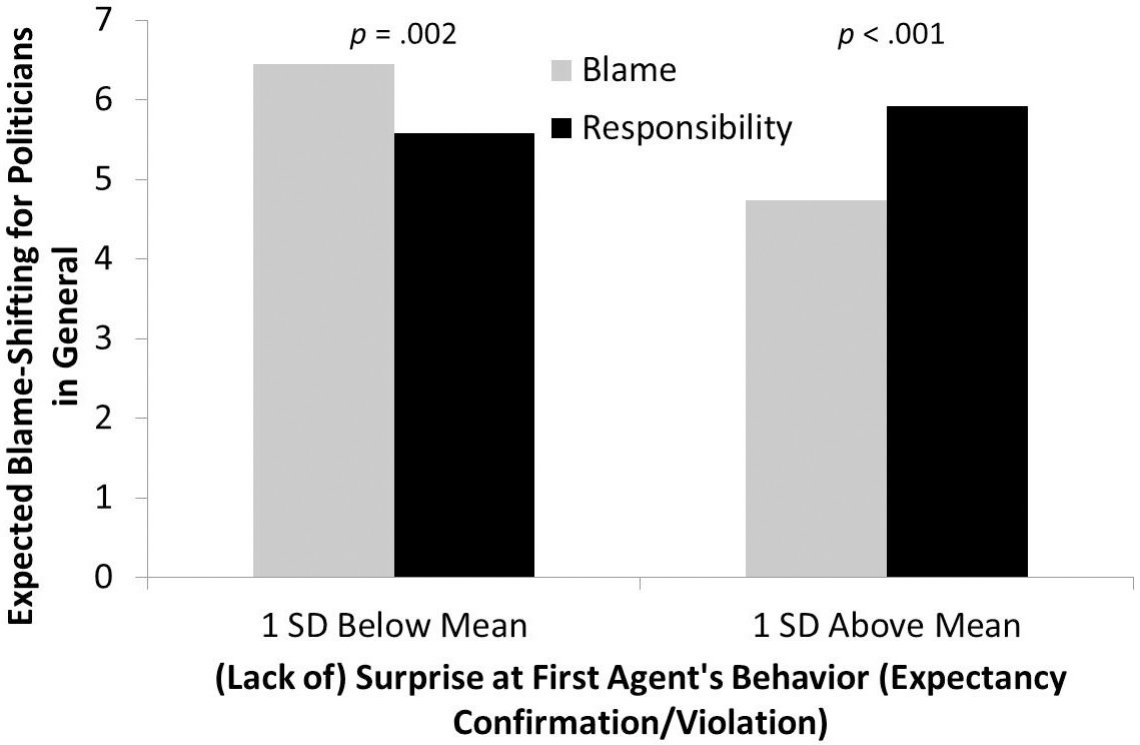
Experiment 4 EBS-politicians. Expected blame-shifting for politicians in general as a function of experimental condition and surprise at first agent’s behavior.

The hypothesized interaction between condition and surprise was significant on EBS-Governor, replicating Experiments 1 and 2, *b* = -0.24, Δ*R*^2^ = .023, *ΔF*(1, 196) = 4.79, *p* = .030. However, even though the pattern of means was the same as in Experiments 1 and 2, the difference between the blame-shifting and responsibility-taking conditions were not significant at 1 *SD* below (*p* = .108) or above (*p* = .130) the mean on surprise. This suggests that condition-based differences at these levels of surprise were somewhat smaller than in the earlier experiments, but would be significant at greater extremes on surprise. The interaction of condition and surprise was not significant for EBS-General, *p* = .702. However, confirming the hypothesized effect in a different way, when using EBS-Politicians—a general measure of the extent to which participants expected politicians to shift the blame—as the dependent variable, the effect was even stronger than for the specific politician participants had read about, *b* = -.65, Δ*R*^2^ = .11, *ΔF*(1, 196) = 23.72, *p* < .001. As can be seen in Fig 3, one *SD* below the mean on surprise, participants in the blame-shifting condition expected significantly more blame-shifting from politicians in general (*p* = .002); at one *SD* above the mean, this reversed, with more surprised participants in the responsibility-taking condition expecting more blame shifting from politicians in general (*p* < .001).

Overall, the results of Experiment 4 were mostly consistent with the results of Experiments 1 and 2, with a few differences. First, there was no main effect of condition on expected blame shifting for the agent in the second story, people in general, or politicians in general. However, the hypothesized interaction of condition and surprise was found again here, even if the effect was somewhat smaller than in earlier experiments. Perhaps most telling and consistent with our hypothesis, a strong interaction between condition and surprised emerged for the extent to which people expected politicians *in general* to shift blame. That is, when participants were unsurprised by a politician shifting blame versus taking responsibility, they continued to expect the same kind of behavior from other, unspecified politicians. However, when they were surprised, they expected *less* blame shifting from other politicians after exposure to an agent who shifted blame and *more* blame shifting when they were surprised by a politician who took responsibility.

## General discussion

Research on blame has received substantial attention, providing a wealth of insight into when, how, and why people blame (e.g., [2–9]). Yet, despite knowing a lot about how blame “works” in forming social and moral evaluations (e.g., [2, 4, 6–9]), we know almost nothing about whether *observing* people casting blame has consequences, suggesting that research on the topic is sorely needed. Of course, in many cases blame is warranted and is used instrumentally to highlight and socially punish actual wrongdoing [7]; in these cases, observing blame may not be particularly consequential, aside from directing people’s attention toward a person who is responsible for causing harm and explaining why harm occurred.

However, in other cases blame is used as a tool to unconscionably deflect criticism away from the self and avoid censure. Although this strategy may be understandable because people have a strong desire to avoid being judged negatively by others [13], if the present work and work by others [1, 36] is any guide, the strategy can backfire. People don’t like it when others shift blame for their own failings. Nevertheless, certain types of people in the public eye—such as business leaders and politicians—may habitually shift blame because taking responsibility could mean the loss of their livelihood. For example, CEO’s who acknowledge their accountability for failure might be fired from their high-paying positions [37], and politicians who fail to uphold campaign promises might lose their next elections [38]. The present research, building on past work [1], used this as a starting point. If certain sorts of people publicly shift blame for their failures, what types of consequences might that have for observers? Specifically, we wanted to test whether observing others shifting blame would not only make observers more likely to shift blame [1], but whether it would make observers more likely to expect *others* to do the same.

What we found did not directly support this hypothesis. Instead, the evidence we found in three experiments is consistent with a different hypothesis: People expect corporate leaders and politicians to shift blame so much that surprising them with evidence to the contrary only makes them expect it *more*. When particularly surprised, blame shifting is particularly expected. On the other hand, when unsurprised at others’ behavior, people simply expect more of the same. In sum, even though people don’t believe it is appropriate to shift blame [1] and don’t really like people who do (Experiments 2 and 4), they nevertheless expect some people to behave in this way (Experiments 3a and 3b), and it seems to be difficult to get people to believe otherwise.

Of course, this work is not without limitations, the most obvious being that what we found is opposite from what we originally predicted. Yet, our confidence is increased by the replication of the effect to a different second agent who is dissimilar from the first (with the exception that both were high-status agents), and a second replication using a new set of targets, with a very similar pattern of means across all experiments.

Another limitation directly related to the first is that our revised hypothesis was necessarily grounded in theory that was developed after the fact. That is, rather than presenting our findings as having been theoretically derived and fully anticipated, we presented the course of this work as transparently as possible, taking care to replicate the finding before attempting to disseminate it. Because of how this work progressed, we acknowledge that further theory development might be needed. Yet, we also believe that the simple case we have laid out—that surprise at a violated expectancy for one sort of behavior can lead to greater expectation of that same behavior for a similar target responding to a similar situation—may not be that far off the mark, and that future research might help to bolster some of the claims that were made.

The present work also raises a number of interesting questions that might be fruitfully investigated in future research. For example, were the responsibility-taking agents in the present research in fact subtyped as deviants in order to maintain stereotypes about how powerful people will respond to failure, and if so, might the effects we found here generalize to other groups where strong stereotypes exist? Will the effect generalize beyond blame shifting to expectations that agents will take credit for themselves and be stingy with praise for others who have helped them achieve their successes? Are expectations for blame-shifting so high that short of full acceptance of responsibility for a failure, people will believe agents are shifting blame even when they are not? Also, because expectations for blame-shifting are high for people from some groups, do people assume that when these agents take responsibility for failures, they are pragmatically trying to gain moral credentials and inspire trust in order to continue on in their high-status positions (i.e., is taking responsibility seen as an impression-management strategy rather than a morally praiseworthy behavior)? The current findings suggest that these questions might represent other promising areas for further research.

Ultimately, this work is only an initial foray into an aspect of blame that has not received much attention: How does exposure to people who blame others (or not) for their own misdeeds affect perceivers’ judgments of other social targets? Given the frequency with which people *do* blame others, the effects of observing this type of behavior on subsequent judgments seems worthy of consideration. Perhaps more important, it raises other intriguing questions about how people think about blame and the extent to which people use blame shifting as a means of self-preservation.
